# *C9orf72* Intermediate Repeats Confer Genetic Risk for Severe COVID-19 Pneumonia Independently of Age

**DOI:** 10.3390/ijms22136991

**Published:** 2021-06-29

**Authors:** Isabella Zanella, Eliana Zacchi, Simone Piva, Massimiliano Filosto, Giada Beligni, Diana Alaverdian, Sara Amitrano, Francesca Fava, Margherita Baldassarri, Elisa Frullanti, Ilaria Meloni, Alessandra Renieri, GEN-COVID Multicenter Study, GEVACOBA Study Group, Francesco Castelli, Eugenia Quiros-Roldan

**Affiliations:** 1 Department of Molecular and Translational Medicine, University of Brescia, 25123 Brescia, Italy; 2 Clinical Chemistry Laboratory, Cytogenetics and Molecular Genetics Section, Diagnostic Department, ASST Spedali Civili di Brescia, 25123 Brescia, Italy; 3 Department of Clinical and Experimental Sciences, University of Brescia, 25123 Brescia, Italy; e.zacchi@studenti.unibs.it (E.Z.); massimiliano.filosto@unibs.it (M.F.); francesco.castelli@unibs.it (F.C.); maria.quirosroldan@unibs.it (E.Q.-R.); 4 Division of Infectious and Tropical Diseases, ASST Spedali Civili di Brescia, 25123 Brescia, Italy; 5 University Division of Anesthesiology and Critical Care Medicine, ASST Spedali Civili, 25123 Brescia, Italy; simone.piva@unibs.it; 6 Department of Medical and Surgical Specialties, Radiological Sciences and Public Health, University of Brescia, 25123 Brescia, Italy; 7 NeMo-Brescia Clinical Center for Neuromuscular Diseases, 25064 Gussago, Italy; 8 Medical Genetics, Department of Medical Biotechnologies, University of Siena, 53100 Siena, Italy; giada.beligni@dbm.unisi.it (G.B.); diana.alaverdian@dbm.unisi.it (D.A.); francesca.fava@dbm.unisi.it (F.F.); margherita.baldassarri@dbm.unisi.it (M.B.); elisa.frullanti@dbm.unisi.it (E.F.); ilaria.meloni@dbm.unisi.it (I.M.); alessandra.renieri@unisi.it (A.R.); 9 Department of Medical Biotechnologies, Med Biotech Hub and Competence Center, University of Siena, 53100 Siena, Italy; 10 Genetica Medica, Azienda Ospedaliera Universitaria Senese, 53100 Siena, Italy; sara.amitrano@unisi.it

**Keywords:** autophagy, COVID-19, *C9orf72*, intermediate alleles, genetic risk, innate immunity, SARS-CoV-2

## Abstract

A cytokine storm, autoimmune features and dysfunctions of myeloid cells significantly contribute to severe coronavirus disease 2019 (COVID-19), caused by the severe acute respiratory syndrome coronavirus 2 (SARS-CoV-2) infection. Genetic background of the host seems to be partly responsible for severe phenotype and genes related to innate immune response seem critical host determinants. The *C9orf72* gene has a role in vesicular trafficking, autophagy regulation and lysosome functions, is highly expressed in myeloid cells and is involved in immune functions, regulating the lysosomal degradation of mediators of innate immunity. A large non-coding hexanucleotide repeat expansion (HRE) in this gene is the main genetic cause of frontotemporal dementia (FTD) and amyotrophic lateral sclerosis (ALS), both characterized by neuroinflammation and high systemic levels of proinflammatory cytokines, while HREs of intermediate length, although rare, are more frequent in autoimmune disorders. *C9orf72* full mutation results in haploinsufficiency and intermediate HREs seem to modulate gene expression as well and impair autophagy. Herein, we sought to explore whether intermediate HREs in *C9orf72* may be a risk factor for severe COVID-19. Although we found intermediate HREs in only a small portion of 240 patients with severe COVID-19 pneumonia, the magnitude of risk for requiring non-invasive or mechanical ventilation conferred by harboring intermediate repeats >10 units in at least one *C9orf72* allele was more than twice respect to having shorter expansions, when adjusted for age (odds ratio (OR) 2.36; 95% confidence interval (CI) 1.04–5.37, *p* = 0.040). The association between intermediate repeats >10 units and more severe clinical outcome (*p* = 0.025) was also validated in an independent cohort of 201 SARS-CoV-2 infected patients. These data suggest that *C9orf72* HREs >10 units may influence the pathogenic process driving more severe COVID-19 phenotypes.

## 1. Introduction

A large non-coding hexanucleotide repeat expansion (HRE) in the *C9orf72* gene (>30 up to 1000 units) is the main genetic cause of frontotemporal dementia (FTD) and amyotrophic lateral sclerosis (ALS) [1,2], both characterized by neuroinflammation and high systemic levels of interleukin-6, interleukin-1β and tumor necrosis factor-α [3]. Healthy people harbor alleles ranging from 2 to 30 repeat units, but a real cut-off has not been determined and HREs of intermediate length (9–30 units), although rare, seem to be more frequent in neurodegenerative, neuropsychiatric and autoimmune disorders [4,5,6,7,8,9,10,11,12]. Gain of functions linked to the presence of the large HRE, resulting in nuclear RNA foci and cytoplasmic aggregation of dipeptide repeat proteins, are the main pathogenic mechanisms of neurodegeneration in FTD and ALS, but *C9orf72* haploinsufficiency is assumed to play a role in the underlying neuroinflammation [13].

The *C9orf72* gene is involved in vesicular trafficking, autophagy regulation and lysosome functions [13]. C9orf72 protein forms a complex with the Smith–Magenis chromosome region candidate 8 (SMCR8) and WD repeat domain 41 (WDR41) proteins [14]. Both C9orf72 and SMCR8 seem to act as guanine-nucleotide (GDP-GTP) exchange factors for several Rab GTPases, a family of proteins each of which localizes on a specific type of cellular membrane compartment and organelle and is involved in trafficking of specific vesicles. Interacting with Rab5, Rab7, Rab11 and Rab7L1, the C9orf72-SMCR8-WDR41 complex regulates the endocytic trafficking and release of extracellular vesicles, while engaged by Rab1, Rab8, Rab39, Rab5 and Rab7 the complex is involved in recruiting the unc-51 like autophagy activating kinase 1 (ULK1) to the phagophore to initiate autophagy, in delivery of cargos to autophagosomes, autophagosome closure and fusion with lysosomes [15,16,17,18,19]. The C9orf72-SMCR8-WDR41 complex also regulates lysosome acidification, regeneration and exocytosis [20,21,22,23,24].

*C9orf72* is ubiquitously expressed in the body with highest levels in myeloid cells. This gene is also differentially expressed with regard to the type of transcript among the 3 described variants and the use of differential transcription start sites for each transcript variant in the brain and myeloid cells, suggesting cell and/or tissue specific functions [25]. Considering the crucial role of autophagy in inflammation and immunity [26], these observations opened the possibility that *C9orf72* loss of function might affect not only neurons but also the innate immune system [25]. Complete loss of the gene in *C9orf72*^−/−^ knock-out mice results in the release of proinflammatory cytokines, splenomegaly, lymphadenopathy and production of autoantibodies, indicating the appearance of autoinflammation and autoimmunity [27,28,29]. Importantly, even hemizygous *C9orf72*^+/−^ mice show altered inflammatory response, suggesting that also haploinsufficiency could lead to unbalanced immunity in mice. More recent work has corroborated these findings, showing that a defective C9orf72-SMCR8-WDR41 complex in murine myeloid cells causes prolonged Toll-like receptor (TLR) signaling and hyperactive type I interferon (IFN) response, due to the disrupted degradation of stimulator of IFN response cGAMP interactor 1 (STING) [30,31].

As stated above, *C9orf72* full mutation results in haploinsufficiency, observed in blood cells and post-mortem brains and spinal cord of ALS/FTD patients [1,32]. *C9orf72* HREs of intermediate length also seem to modulate gene expression. Expansions of more than 8 repeats mainly occur within a 110 kb FTD/ALS risk haplotype, that is more common in individuals of Northern European ancestry [33]. This haplotype was found associated with slightly higher expression of *C9orf72* transcript variants 1 and 3 (both having the HRE region within intron 1) and lower expression of the most abundant transcript variant 2 (with the HRE located in the promoter), with more marked effect in the case of homozygosity for the risk haplotype [25]. Similar findings were described by Cali and colleagues [10], who found the risk alleles significantly associated with increased *C9orf72* expression across several tissues, with the largest effect in neural tissues. The same authors also demonstrated the increase in transcript variant 3 and protein levels in induced pluripotent stem cells edited with intermediate HREs and differentiated into neural progenitor cells [10]. In contrast, Gijselinck and colleagues [34] found that repeat length from 7 to 24 unit resulted in slightly higher methylation degree in comparison with shorter repeats in humans, particularly in the homozygous state, and observed a decrease of *C9orf72* promoter transcriptional activity with increasing number of repeats from 7 to 24 units in HEK293T and SH-SY5Y cells.

Both decrease and increase of *C9orf72* expression have been found to impair autophagy [10,35,36]. We hypothesized that this effect may also reflect in host immune response to infections and that harboring *C9orf72* HREs of intermediate length may then modulate this response. It has been recently found that gut microbiota may influence the autoinflammatory phenotype of *C9orf72*^−/−^ mice [37] and that Herpes Simplex Virus-2 (HSV-2) infection in spinal cord of mice results in the decrease of C9orf72 protein [38]. Apart from the above observations and the described role of C9orf72 in regulating TLR and type I IFN signaling in mice, there is no evidence of an involvement of the gene in host response to infectious diseases in humans.

Since 2020, we have faced the coronavirus disease 2019 (COVID-19) pandemic caused by the severe acute respiratory syndrome coronavirus 2 (SARS-CoV-2) infection. Despite enormous efforts of the scientific community, there is still a lack of knowledge on the pathogenic mechanisms of this new virus. Excessive inflammation, autoimmune phenomena and defective antiviral type I IFN signaling are believed to significantly contribute to COVID-19 severity [39,40,41,42,43]. Genetic background contributes to susceptibility to autoimmune and infectious diseases in humans and genetic variants associated with those diseases are often found in genes involved in immune response and inflammation, including genes related to the autophagy pathways [44,45]. Furthermore, a multifactorial risk score for COVID-19 severity based on a polygenic model and including autophagy genes has been recently proposed [46].

In view of the above, in the present study we have explored the hypothesis that normal, but in the upper range, HREs in the *C9orf72* gene could represent a risk factor for the development of more severe COVID-19 forms.

## 2. Results

In the present study, we initially included 240 patients with severe COVID-19 defined by SARS-CoV-2 positive molecular test and pneumonia that requires hospitalization. During hospitalization, 92 out of 240 (38.3%) patients received mechanical ventilation (MV) or non-invasive ventilation (NIV). Need of MV or NIV was used to define the most severe degree of COVID-19 in further analyses.

In order to explore our hypothesis, we compared *C9orf72* repeat size, allele distribution and frequency with those observed in a historical cohort of genetically characterized patients with ALS (*n* = 93), harboring no *C9orf72* pathogenic large expansions, without clinically defined disorders related to immune dysfunctions, mostly Caucasian and from the same geographical region (Lombardy, Italy) of COVID-19 patients. Indeed, no significant differences in the distribution of repeat size, allele distribution and frequency have been observed between *C9orf72* expansion-negative ALS cases and healthy controls in published studies [1,2,4,8,47,48,49,50], while the length of the HRE may depend on the genetic ancestry, being expansions of more than 8 repeats linked to the chromosome 9 Finnish founder ALS risk haplotype that is common in individuals of Northern Europe ancestry [33]. Therefore, the cohort of *C9orf72* expansion-negative ALS patients from the same geographical area of COVID-19 patients can be considered as representative of the population of that region regarding the genetic background relative to the *C9orf72* gene and used to compare COVID-19 patients. Demographic data of all patients are described in Table 1. No differences in sex, age and ethnicity were found between the two sub-cohorts.

**Figure 1:**
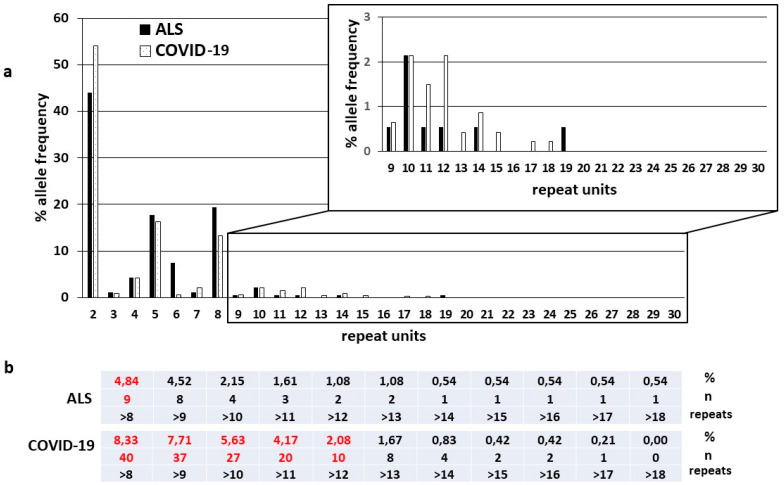
(**a**) Histogram showing the distribution and frequency (%) (Y-axis) of the number of *C9orf72* hexanucleotide repeats (X-axis) in ALS (black bars) and COVID-19 (white bars) patients’ alleles. (**b**) Frequency (%) and number (*n*) of intermediate alleles for each repeat length >8 repeat units in ALS and COVID-19 patients. Both for ALS and COVID-19 patients, the % and n for repeat length > 8 units are in red; for COVID-19 patients, % and n with a clear difference in comparison with ALS patients are also highlighted in red.

Genetic analysis for *C9orf72* HREs in the 240 COVID-19 patients did not reveal the presence of large (>30 repeats) expansions. Alleles with 2, 5, and 8 repeat units were the most frequent HREs in both sub-cohorts (Figure 1), as previously reported in several populations of both ALS patients and healthy controls [1,2,4,5,6,7,8,9,10,11,12,47,48,49,50].

Based on a preliminary size cut-off of >8 and ≤30 repeat units to define intermediate lengths, which was determined on the basis of previous studies [12] (see Materials and Methods section for more details), we found *C9orf72* intermediate HREs in 39 out of 240 (16.25%) hospitalized COVID-19 patients and 8 out of 93 (8.60%) ALS patients, with a trend towards a higher prevalence of intermediate expansions in hospitalized COVID-19 vs. ALS patients, despite comparable average, median number and range of repeat units (Table 1). Intermediate HREs were present on both alleles in only one COVID-19 patient and only one ALS patient. Comparing the overall number of intermediate alleles, we then found 40 out of 480 (8.33%) intermediate alleles in hospitalized COVID-19 patients and 9 out of 186 (4.84%) in ALS patients (Figure 1). The ALS patient with both intermediate alleles is an Italian female (age 70 years) that started with spastic dysarthria onset and predominant involvement of motor neuron I and evolved in anarthria as main clinical characteristic. Currently she wears percutaneous endoscopic gastrostomy (PEG) and is employing night-time NIV. The COVID-19 patient with both intermediate alleles is a Caucasian male (age 36 years) with negative anamnesis that during hospitalization received MV and dialysis due to acute immune-mediated glomerular disease and tubular injury.

**Figure 2:**
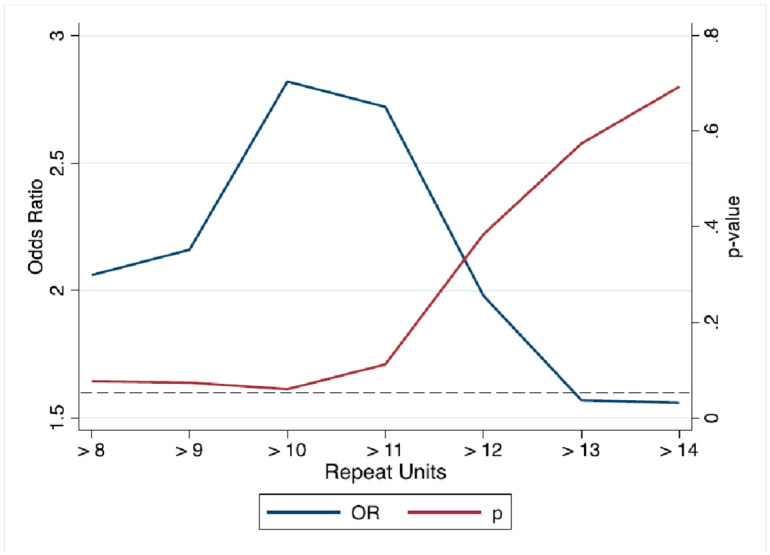
Results of univariate logistic regression analysis between COVID-19 cases and ALS patients at each intermediate allele size > 8 units. The blue line represents odds ratio (OR), the dark red line *p*-value, and the dotted gray line represents *p* = 0.05 significance level.

Considering data shown in Figure 1 and in order to find the number of *C9orf72* hexanucleotide repeats that may better distinguish between COVID-19 and ALS patients, we conducted univariate logistic regression analysis at each repeat length >8 repeat units (Figure 2) and found that patients hospitalized for COVID-19 had an odds ratio (OR) of 2.82 (*p* = 0.06) of having more than 10 repeats, when compared to the ALS patients.

Moreover, 27 out of 240 (11.25%) COVID-19 patients had at least one allele with more than 10 repeats compared to 4 out of 93 (4.30%) ALS patients (*p* = 0.050) (Table 1). Comparing the overall number of intermediate alleles with > 10 repeats, we found 27 out of 480 alleles (5.63%) with more than 10 repeats in COVID-19 patients vs. 4 out of 186 (2.15%) in ALS patients (*p* = 0.056) (Table 1).

Univariate logistic regression analysis (Table 2) reveals that COVID-19 patients with more than 10 repeats in at least one allele are younger than patients with shorter expansions [mean age (±SD) 60.67 (±13.37) vs. 64.76 (±11.98), *p* = 0.090] and required more frequently MV or NIV (56% vs. 36%, *p* = 0.053), although differences did not reach the statistical significance. We also analyzed routine laboratory parameters, however no significant differences in terms of mean values were found, except for D-dimer levels (*p* = 0.02) (Table 2).

Multivariate regression analysis further suggested the presence of more than 10 repeats in at least one allele as a possible risk factor for NIV or MV requirements independently of age in patients with COVID-19 pneumonia (OR 2.36, 95% confidence interval (CI) 1.04–5.37, *p* = 0.040] (Table 3).

Finally, we replicated our analysis in an independent cohort of 201 SARS-CoV-2 infected individuals from the GEN-COVID Multicenter Study [51]. This replication cohort included 101 severely affected COVID-19 patients who received MV or NIV during hospitalization and 100 non-hospitalized subjects (asymptomatic or with very mild symptoms). Demographic data of patients are described in Table 4. As expected, severely affected COVID-19 patients were mostly males and older in comparison with non-hospitalized patients.

As in the first cohort of COVID-19 patients, we did not find large (>30 repeats) *C9orf72* expansions. Based on the results obtained with the first cohort, we chose a cut-off value of more than 10 repeats and stratified patients by disease severity. We found 16 out of 101 (15.84%) subjects with at least one *C9orf72* allele with more than 10 repeats in COVID-19 patients treated by either MV or NIV and 6 out of 100 (6%) in non-hospitalized SARS-CoV-2 infected subjects (*p* = 0.025). In this cohort we did not find subjects with more than 10 repeats in both *C9orf72* alleles. When considering the overall number of alleles, we found 16 out of 202 alleles (7.92%) with more 10 repeats in the first group (patients treated by MV or NIV) and 6 out of 200 (3%) in the second group (asymptomatic or with very mild symptoms) of SARS-CoV-2 infected patients (*p* = 0.030) (Table 4).

**Figure 3:**
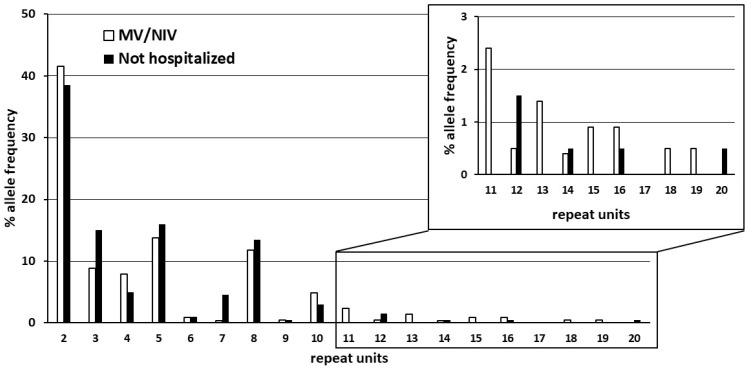
Histogram showing the distribution and frequency (%) (Y-axis) of the number of *C9orf72* hexanucleotide repeats (X-axis) in patients’ alleles in severe COVID-19 patients who received mechanical ventilation (MV) or non-invasive ventilation (NIV) (white bars) and non-hospitalized SARS-CoV-2 infected subjects (asymptomatic or with very mild symptoms) (black bars).

Figure 3 shows the distribution and frequency of the number of *C9orf72* repeats in the validation cohort and highlights alleles with more than 10 repeats. These results confirmed the association of severe COVID-19 that requires MV or NIV with the presence of longer intermediate repeats (>10 units) in the *C9orf72* gene.

## 3. Discussion

With this work, we sought to explore whether intermediate HREs in *C9orf72* may be a risk factor for severe COVID-19 pneumonia. Although we found intermediate repeats in only a small percentage of COVID-19 patients, the magnitude of risk for requiring MV or NIV conferred by harboring intermediate repeats >10 units in at least one allele was more than twice with respect to having shorter expansions (≤10 units), when adjusted for age (OR 2.36; 95% C.I. 1.04–5.37, *p* = 0.040). The association between intermediate repeats >10 units and more severe clinical outcome (*p* = 0.025) was also validated in an independent cohort of 201 SARS-CoV-2 infected patients, comprising 101 severely affected COVID-19 patients who received MV or NIV during hospitalization and, as control group, 100 non-hospitalized subjects (asymptomatic or with very mild symptoms). These data suggest that *C9orf72* HREs > 10 units may be not a common cause of severe COVID-19 pneumonia but may influence the pathogenic process driving to severe phenotypes.

Although mainly implicated in neurodegenerative disorders, the human gene *C9orf72* is highly expressed not only in microglia but also in myeloid cells, mainly monocytes and dendritic cells [25], is critical for their proper functions and is involved in autoimmunity and inflammation [27,28,29]. *C9orf72*^−/−^ knock-out mice indeed exhibit dysregulation of the immune system, age-dependent inflammation characterized by a cytokine storm, neuroinflammation and features of autoimmunity like systemic lymphadenopathy, splenomegaly, pseudothrombocytopenia, high levels of autoantibodies and membrano-proliferative glomerulonephritis reminiscent of systemic lupus erythematosus (SLE). Even haploinsufficient hemyzygous *C9orf72*^+/−^ mice exhibit enhanced cytokine production in response to several immune stimuli [28]. Interestingly, we found that one COVID-19 patient with intermediate HREs in both *C9orf72* alleles received MV during hospitalization and experienced acute immune-mediated glomerular disease.

Accumulating evidence supports the role of *C9orf72* in regulating vesicle trafficking [15,18,52,53] and lysosomal degradation of inflammatory mediators, including TLRs and STING, leading to their prolonged inflammatory signaling [30,31]. Interestingly, the environment, especially variation in gut microorganisms, seems to directly influence the pathological phenotype of *C9orf72*^−/−^ mice [37] and HSV-2 latent infection in the spinal cord of mice results in altered microglia and leucocyte infiltration accompanied by a decrease in C9orf72 protein levels [38]. C9orf72 interacts with different Rab GTPases and might affect autophagy at many steps and through the regulation of mammalian target of rapamycin Complex 1 (mTORC1) [13]. Of note, autophagy dysfunctions are often associated with inflammatory and autoimmune diseases [44] and innate immune responses and inflammation, crucial in anti-viral responses, are regulated by autophagy [54]. Several studies have shown that many viruses, like coronaviruses, have evolved strategies to evade the host response by directly hijacking the autophagy pathway in support of their life cycle and spread or by disrupting the host control on the production of anti-viral cytokines [54,55,56]. Host genetics also contributes to aberrant immunity in autoimmune diseases and susceptibility to infectious diseases in humans and such variants are often found in genes involved in the immune response and inflammation [44,45]. The current knowledge and our work confirm these findings in COVID-19 [41,43,45,57,58]. Autophagy genes have recently been proposed as susceptibility factors in COVID-19 [46]. Our results are the first report on the potential involvement of variants in an autophagy gene in determining susceptibility for severe COVID-19 phenotype. The recent observation that C9orf72 is involved in the lysosomal degradation of inflammatory mediators like TLRs and STING [30,31], that are crucial in anti-viral response, further corroborates our findings.

Large hexanucleotide expansions in *C9orf72* lead to neurodegeneration in ALS/FTD through the cooperation between loss and gain of functions, derived from *C9orf72* haploinsufficiency and accumulation in patients’ brain and spinal cord of *C9orf72* HRE bidirectional transcripts and cytoplasmic toxic aggregates of dipeptide repeat proteins (DPRs) [13]. *C9orf72* intermediate expansions of 24–30 repeats have recently been found associated with ALS in a large meta-analysis on 5071 cases and 3747 controls [59], but characteristic nuclear RNA foci and DPR aggregates were absent in one ALS patient with an intermediate expansion of 16 repeats [60] and 9 cases with corticobasal degeneration and intermediate repeats ranging from 17 to 29 units [10]. Furthermore, while full expansion results in the decrease in *C9orf72* mRNA and protein expression [1,61], due to premature abortion of transcription [62] and hypermethylation of the CpG-rich *C9orf72* promoter region [34,63], for intermediate expansions current results are discordant. Some authors and our group found association of intermediate repeats with neurodegenerative disorders like corticobasal degeneration, Parkinson’s Disease, atypical parkinsonisms, multiple sclerosis, psychiatric symptoms in ALS/FTD patients, neuropsychiatric disorders and autoimmune diseases [4,5,6,7,8,9,10,11,12]. Moreover, intermediate repeats from 7 to 24 units showed a slightly higher methylation degree, particularly in the homozygous state, in comparison with short repeats. A decrease of transcriptional activity with increasing number of repeats from 7 to 24 units compared with shorter repeats has been demonstrated in HEK293T and SH-SY5Y cells [34]. By contrast, the risk haplotype was found to be associated with slightly higher expression of *C9orf72* transcript variants 1 and 3 and lower expression of transcript variant 2 [25] and induced pluripotent stem cells edited with intermediate HREs and differentiated into neural progenitor cells showed an increase in transcript variant 3 and protein levels [10]. As stated above, *C9orf72* protein expression is down-modulated by HSV-2 infection [38] while a cell type-dependent regulation of its levels via the ubiquitin-proteasome system and autophagy has been recently suggested [35]. Given the role of C9orf72 in TLR and type I IFN pathways [30,31], it is tempting to speculate that intermediate repeats, likely through gene expression modulation, may influence host response to infection with SARS-CoV-2 and perhaps further viruses. This could explain our findings regarding the higher risk of having severe COVID-19 requiring NIV or MV independently of age. Indeed, hyperactivation of myeloid cells, aberrant release of pro-inflammatory cytokines, autoimmune features and defective innate immune responses, particularly in type I IFN signaling, are believed to significantly contribute to severe clinical course of COVID-19. Recent studies highlighted the role of host genetics in determining COVID-19 severity with the identification of inborn errors of TLR3, IFN regulator factor 7-dependent production of type I IFN and variants in further genes involved in IFN signaling, cytokine release and inflammation underlying life-threatening COVID-19 [41,43,58]. To date, there is limited direct experimental evidence on autophagy involvement in SARS-CoV-2 infection, either in an anti-viral or pro-viral manner, with the exception of recent studies demonstrating that the SARS-CoV-2 papain-like protease (PLpro) cleaves the serine/threonine kinase unc-51-like autophagy activating kinase 1 disrupting autophagy [64] and that SARS-CoV-2 ORF3a inhibits autophagy activity by blocking fusion of autophagosomes/amphisomes with lysosomes [65]. We can hypothesize that harboring intermediate HREs in *C9orf72* could contribute to negatively balancing the host innate immune response to SARS-Cov-2 infection leading to a more severe disease. A limit of our study is that we did not measure *C9orf72* mRNA expression in patients’ peripheral blood cells, however we thought that gene expression could be influenced not only by harboring intermediate repeats >10 units, as described above [10,25,34], but also by the clinical state, as suggested for HSV-2 infection [38], making it hard to discriminate between the two conditions. COVID-19 patients of the first cohort were enrolled after discharge, however they had been severe COVID-19 hospitalized patients (38.3% of them received MV or NIV) and most of them at the time of the recruitment in this study, during the follow-up, were still showing some signs of severe COVID-19. This could make difficult and hamper a clean analysis of *C9orf72* expression relative to the length of the intermediate expansion. Furthermore, some patients of the validation cohort were recruited during the pandemic and the ongoing inflammatory conditions could likely affect *C9orf72* expression. Further studies are therefore needed to determine if intermediate expansions may modulate *C9orf72* in vivo and, more importantly, which immune cells are mainly affected but also to verify if SARS-CoV-2 may influence *C9orf72* expression in particular subsets of myeloid cells.

Increased levels of pro-inflammatory cytokines have been observed in sera of *C9orf72*^−/−^ knockout mice [27,28,29,37], with a pattern that similarly defines the “cytokine storm” driving acute injuries during severe COVID-19 [66]. In our cohort of severe COVID-19 patients, we did not find any evident correlation between the presence of *C9orf72* intermediate repeats and routine inflammatory laboratory parameters, except for D-dimers, and we did not measure levels of pro-inflammatory chemokines and cytokines. This is a limit of our study. Coagulation biomarkers, including D-dimers, are frequently altered during severe inflammation [67,68,69,70]. In patients with severe COVID-19, genetic variants studied here may be involved in more severe inflammatory conditions perhaps through STING signaling-mediated altered type I IFN production [31]. Indeed, very recently, inflammasome-dependent coagulation activation has been found to associate with excessive activation of the STING pathway [67], while beclin-1, a marker of autophagy, has been found to be increased in COVID-19 patients, particularly in severe patients, and its levels have been demonstrated to correlate with D-dimer levels [71].

The complex interactions between genetic background and the environment are poorly understood. The variable phenotype associated with *C9orf72* large HREs in ALS/FTD has indicated that penetrance is incomplete [72], suggesting that either further genetic or environmental factors could modify the individual risk of disease. Microbiota seems to be a potent modifier of onset and progression of autoimmunity, inflammation and premature mortality in *C9orf72*^−/−^ knockout mice [37]. We cannot, then, exclude that environmental factors like microbiota may also influence the effect of intermediate *C9orf72* repeats on COVID-19 clinical phenotype.

Further limitations of our study are, first, that the number of carriers of *C9orf72* intermediate alleles in the 240 severe COVID-19 patient cohort, as well as in the validation cohort of 201 SARS-CoV-2 infected patients, is small, and one should be cautious with the interpretation of these results. Secondly, we considered a cohort of genetically characterized patients with ALS, harboring no *C9orf72* pathogenic large expansions and without clinically defined disorders related to immune dysfunctions, as representative of the general population for the first part of this study rather than considering uninfected controls, likely resistant to SARS-CoV-2 infection, to make the first comparisons and find the number of repeats in *C9orf72* HRE at which the difference between COVID-19 patients and ALS patients was significant. Nevertheless, at the time of patients’ recruitment for this study we had no easy access to SARS-CoV-2 negative subjects, since in the midst of the pandemic molecular tests were executed mainly in symptomatic patients in Italy and, however, we could have not been sure that SARS-CoV-2 negative subjects were not infected because of their genetic background or simply because they did not come into close contact with infected people. Therefore, SARS-CoV-2 negative subjects could not represent the correct control population. Furthermore, as stated above, all published studies performed in ALS cases without pathological *C9orf72* expansions and healthy controls found no significant differences in distribution, range, and median number of repeats [1,2,8,47,48]. For these reasons, and to avoid bias possibly deriving from genetic ancestry, being expansions of more than 8 repeats linked to the chromosome 9 Finnish founder ALS risk haplotype that is more common in individuals of Northern Europe ancestry [33], we decided to choose the ALS cohort (mostly Caucasian and from the same geographical region of COVID-19 patients), already used in a previous work [12] for the first comparison. Moreover, further analyses in this work compared severe COVID-19 patients of the first cohort considering MV and NIV requirement as a proxy of high severity of disease to find association with *C9orf72* intermediate repeats >10 units. Furthermore, we validated our findings in an additional cohort. Since the aim of the second part of our work was not the comparison between COVID-19 patients and the general population but the confirmation of our hypothesis that harboring alleles with more than 10 repeats in the *C9orf72* gene may be a risk to develop a more severe form of disease, we considered only COVID-19 patients stratified in severely affected ones that received MV or NIV during hospitalization and non-hospitalized subjects (asymptomatic or with very mild symptoms). The genetic analyses in this stratified cohort confirmed the association between intermediate repeats >10 units and more severe clinical outcome.

Finally, we cannot completely exclude that COVID-19 severity could be unrelated to *C9orf72* HRE itself but rather associate with the genetic background defined by the chromosome 9 region in which *C9orf72* is located, comprising the 110 kb risk Finnish haplotype, that is, as stated above, more frequent for alleles with more than 8 repeats within the *C9orf72* HRE. Interestingly, genome-wide association studies (GWAS) identified single nucleotide polymorphisms (SNPs) in the region of chromosome 9 that contains the Mps One Binder Kinase Activator-Like 2B (MOBKL2B), *C9orf72* and IFN-K loci as associated with the response to anti-tumor necrosis factor α therapy in rheumatoid arthritis (RA) [73], with a genetic predisposition to SLE [74] and recently as genetic loci shared between ALS and autoimmune diseases like SLE and RA [75]. IFN-K is expressed in oral epithelial cells, one of the first sites of host interaction with viruses that are spread via saliva and may be spread through the mouth [76]. Near this region are also clustered further genes of type I IFNs and we recently found a significantly higher frequency of *C9orf72* intermediate repeats in patients with SLE and RA [12].

In conclusion, *C9orf72* intermediate alleles >10 repeat units are over-represented in hospitalized COVID-19 patients with severe pneumonia and related to MV and NIV requirements independently of age, suggesting that they could represent a risk factor contributing to the occurrence of severe COVID-19 forms. Autophagy may be involved in the COVID-19 clinical phenotype and a polygenic model also related to genes involved in the autophagy machinery has been recently proposed to explain COVID-19 risk assessment and guide precision medical care [46]. This is the first report describing the association of severe forms of COVID-19 with variants in a gene involved in autophagy. Understanding how host genetic factors contribute to variation in disease susceptibility and severity may shed light on heterogeneity in the immune response and the host–pathogen interaction and facilitate the development of therapeutics and vaccines.

## 4. Materials and Methods

### 4.1. Patients

In the first cohort, we consecutively enrolled 240 adult patients (aged > 18 years, mostly Caucasian and most of them from the same Italian region, Lombardy) with confirmed COVID-19 pneumonia (defined by SARS-CoV-2-positive molecular test on nasopharyngeal swab and radiological features of pneumonia) who previously required hospitalization at ASST-Spedali Civili di Brescia over the period March-December 2020. Recruitment was performed when discharged patients were referred to the University Department of Infectious and Tropical Diseases of our Hospital for clinical and virological control and follow-up. Hospitalization with COVID-19 pneumonia was used as proxy of severity for patients’ inclusion. NIV or MV were used to define the most severe degree of pneumonia in further analyses. Patients’ clinical data and routine laboratory findings (white blood cell, lymphocyte and platelet counts, serum biochemical tests for liver and renal function, C-reactive protein, ferritin, D-dimer) were collected from clinical and electronic charts. The worst value for each biochemical parameter during hospitalization was used for analyses.

No significant differences between ALS cases without large *C9orf72* HRE and healthy subjects have been thoroughly described regarding distribution, range and median repeat number [1,2,4,8,47,48,49,50]. Nonetheless, differences in the prevalence of large *C9orf72* pathogenic expansions have been described between people from Southern and Northern Europe and both large (>30 repeat units) and intermediate (>8 but ≤30 repeat units) expansions are linked to the chromosome 9 Finnish founder ALS risk haplotype, that is common in individuals of Northern European ancestry [33]. To avoid potential bias deriving from the genetic background, as control group for the first cohort of analyzed subjects we included 93 ALS patients, mostly Caucasian and from the same geographical region of COVID-19 patients, but without large *C9orf72* pathogenic expansions. ALS patients included in this study referred to the Centre for Neuromuscular Diseases and Neuropathies ASST-Spedali Civili di Brescia and were recently admitted to the Cytogenetics and Molecular Genetics Section of our Hospital for routine genetic diagnosis (some of these patients were already described in reference [12].

In the replication study, 201 SARS-CoV-2 infected individuals (defined by SARS-CoV-2-positive molecular test on nasopharyngeal swab as above) from the GEN-COVID Multicenter Study [51] were considered. Among them, 101 patients were severely affected and treated by either MV or NIV, while 100 were non-hospitalized subjects (asymptomatic or with very mild symptoms). Specimens were provided by the COVID-19 Biobank of Siena, which is part of the Genetic Biobank of Siena, a member of BBMRI-IT, Telethon Network of Genetic Biobanks (project no. GTB18001), EuroBioBank and RD-Connect.

All data were collected in anonymized form by study physicians. Written informed consent was obtained by all patients. The protocol for enrollment of COVID-19 patients of the first cohort was approved by the Ethics Committee of ASST-Spedali Civili di Brescia (GEVACOBA Study Project). The GEN-COVID study was approved by the University Hospital of Siena Ethics Review Board. Clinical research was conducted in accordance with the principles for medical research involving human subjects described in the Declaration of Helsinki.

### 4.2. C9orf72 Genotyping

Genomic DNA samples were obtained from peripheral blood samples using the Wizard Genomic DNA Purification kit (Promega Corporation, Madison, WI, USA). DNA samples were quantified by the use of Qubit 2.0 Fluorometer (Thermo Fisher Scientific, Waltham, MA, USA), with Qubit dsDNA HS Assay Kit (Thermo Fisher Scientific) and genotyped with a polymerase chain reaction (PCR)-based two-step *C9orf72* analysis, essentially as previously described [77].

A preliminary cut-off of >8 repeat units was chosen to distinguish short (2–8 units) from intermediate (9–30 units) *C9orf72* HREs, on the basis of the following criteria [12]: (1) most healthy individuals harbor 2 to 8 repeats [1,2]; (2) the risk haplotype is more frequent above 8 repeats [78,79]; (3) modulation of *C9orf72* expression has been observed with a number of repeats in the intermediate range [10,25,34].

### 4.3. Statistics

Categorical variables were reported as proportion and/or percentage, continuous variables as mean (±SD) values. Fisher’s exact or Chi-square test for categorical variables and Student’s *t*-test for continuous variables were applied as appropriate. To find the number of repeats in *C9orf72* HRE at which the differences in COVID-19 and control patients was more significant, we performed a logistic regression analysis, using the COVID-19 condition as dependent variable and the number of patients with different maximum repeats level. We then plotted the OR and *p*-value on the number of maximum repeats.

Logistic regression was used to perform the adjusted analysis for COVID-19 severity (using NIV and MV requirements as proxy), and presence of *C9orf72* HRE >10 units, adjusted for age. *p* values < 0.05 were considered significant. When significant, OR with 95% CI were indicated.

## Figures and Tables

**Table 1:** Demographic data and *C9orf72* hexanucleotide expansions in the cohort of 240 coronavirus disease 2019 (COVID-19) and 93 amyotrophic lateral sclerosis (ALS) patients.

	COVID-19 (*n* = 240)	ALS (*n* = 93)	*p* *
Sex, female (%)	147 (61.25 %)	55 (59.13 %)	0.125
Mean age ± SD	64.30 ± 12.18	64.74 ± 13.22	0.774
Age range	30–95	33–89	
Caucasian ethnicity	230 (95.83%)	91 (97.85%)	0.376
Average number of repeats ± SD	4.37 ± 3.15	4.61 ± 2.89	0.378
Median number of repeats [interquartile range]	2 [2–6]	5 [2–7]	
Range	2–18	2–19	
Patients with >10 repeats (%)	27 (11.25%)	4 (4.30%)	0.050
Alleles with >10 repeats (%)	27 (5.63%)	4 (2.15%)	0.056

SD: standard deviation. * Fisher’s exact test or Chi-square test for categorical variables and Student’s *t*-test for continuous variables were applied as appropriate.

**Table 2:** Univariate regression analysis for the considered parameters in the severe COVID-19 sub-cohort (*n* = 240) with >10 or ≤10 repeats in the *C9orf72* hexanucleotide repeat expansion (HRE) region.

	Normal Values	≤10 Repeats (*n* = 213)	>10 Repeats (*n* = 27)	*p*
Age, year Mean (±SD)		64.76 (± 11.98)	60.67 (± 13.37)	0.090
AST, U/L Mean (±SD)	10–50	79.73 (± 79.88)	73.40 (± 46.01)	0.690
ALT, U/L Mean (±SD)	18–39	74.60 (±94.05)	59.913 (±29.86)	0.458
Ferritin, ug/L Mean (±SD)	30–400	1334.82 (±1541.88)	1125.73 (±756.03)	0.525
Creatinine, mg/dL Mean (±SD)	0.7–1.2	1.40 (±1.35)	1.54 (±2.33)	0.651
Urea, mg/dL Mean (±SD)	17–49	75.97 (±6.06)	101.00 (±27.67)	0.228
D-dimer, ng/mL Mean (± SD)	<232	1623.84 (±290.31)	4552.09 (±2753.16)	0.020
Albumin, g/L Mean (±SD)	31.0–52.0	32.70 (±0.41)	33.24 (±0.90)	0.649
WBC, ×10^3^/uL Mean (±SD)	4.0–10.8	14.32 (±46.77)	10.28 (±6.27)	0.679
Lymphocytes, ×10^3^/uL Mean (±SD)	0.9–4.0	1.94 (±0.16)	1.82 (±0.24)	0.791
CRP, mg/L Mean (±SD)	<5.0	88.71 (±79.32)	71.78 (±49.39)	0.299
Platelets, ×10^3^/uL Mean (±SD)	130–400	343.01(±162.10)	338.10 (±144.42)	0.889
MV or NIV, *n* (%)		77/213 (36%)	15/27 (56%)	0.053

SD: standard deviation; ALT: alanine aminotransferase; AST: aspartate aminotransferase; WBC: white blood cells; CRP: C-reactive protein; MV: mechanical ventilation; NIV: non-invasive ventilation.

**Table 3:** Multivariate regression analysis for the probability to receive invasive mechanical ventilation (MV) or non-invasive ventilation (NIV).

Variables	OR (95% CI)	*P*
Repeats > 10	2.36 (1.04–5.37)	0.040
Age, years	1.01 (0.99–1.04)	0.142

OR, odds ratio; CI, confidence interval.

**Table 4:** Demographic data and *C9orf72* hexanucleotide expansions in the validation cohort.

	MV/NIV (*n* = 101)	Non-Hospitalized (*n* = 100)	*p* *
Sex, female (%)	23 (22.77%)	49 (49%)	0.0001
Mean age ± SD	63.94 ± 9.07	51.97 ± 12.17	0.0001
Age range	33–86	18–81	
Caucasian ethnicity	97 (96.03%)	100 (100%)	0.1213
Average number of repeats ± SD	4.86 ± 3.64	4.47± 3	0.408
Median number of repeats [interquartile range]	3 [2–8]	3 [2–6]	
Range	2–19	2–20	
Patients with >10 repeats (%)	16 (15,84%)	6 (6%)	0.025
Alleles with >10 repeats (%)	16 (7.92%)	6 (3%)	0.030

SD: standard deviation. * Fisher’s exact test or Chi-square test for categorical variables and Student’s *t*-test for continuous variables were applied as appropriate.

## Data Availability

All study data, including raw and analyzed data, and materials will be available from the corresponding author on reasonable request.

## Appendix A

**Collaborators of GEN-COVID Multicenter Study** (https://sites.google.com/dbm.unisi.it/gen-covid) (Accessed on 28 June 2021)
  Francesca Mari (Med Biotech Hub and Competence Center, Department of Medical Biotechnologies, University of Siena, Italy; Medical Genetics, Department of Medical Biotechnologies, University of Siena, Italy; Genetica Medica, Azienda Ospedaliero-Universitaria Senese, Italy), Sergio Daga (Med Biotech Hub and Competence Center, Department of Medical Biotechnologies, University of Siena, Italy; Medical Genetics, Department of Medical Biotechnologies, University of Siena, Italy), Elisa Benetti (Med Biotech Hub and Competence Center, Department of Medical Biotechnologies), Simone Furini (Med Biotech Hub and Competence Center, Department of Medical Biotechnologies), Chiara Fallerini (Med Biotech Hub and Competence Center, Department of Medical Biotechnologies, University of Siena, Italy; Medical Genetics, Department of Medical Biotechnologies, University of Siena, Italy), Floriana Valentino (Med Biotech Hub and Competence Center, Department of Medical Biotechnologies, University of Siena, Italy; Medical Genetics, Department of Medical Biotechnologies, University of Siena, Italy), Gabriella Doddato (Med Biotech Hub and Competence Center, Department of Medical Biotechnologies, University of Siena, Italy; Medical Genetics, Department of Medical Biotechnologies, University of Siena, Italy), Annarita Giliberti (Med Biotech Hub and Competence Center, Department of Medical Biotechnologies, University of Siena, Italy; Medical Genetics, Department of Medical Biotechnologies, University of Siena, Italy), Rossella Tita (Genetica Medica, Azienda Ospedaliero-Universitaria Senese, Italy), Mirella Bruttini (Med Biotech Hub and Competence Center, Department of Medical Biotechnologies, University of Siena, Italy; Medical Genetics, Department of Medical Biotechnologies, University of Siena, Italy; Genetica Medica, Azienda Ospedaliero-Universitaria Senese, Italy), Susanna Croci (Med Biotech Hub and Competence Center, Department of Medical Biotechnologies, University of Siena, Italy; Medical Genetics, Department of Medical Biotechnologies, University of Siena, Italy), Anna Maria Pinto (Genetica Medica, Azienda Ospedaliero-Universitaria Senese, Italy), Maria Antonietta Mencarelli (Genetica Medica, Azienda Ospedaliero-Universitaria Senese, Italy), Caterina Lo Rizzo (Genetica Medica, Azienda Ospedaliero-Universitaria Senese, Italy), Francesca Montagnani (Med Biotech Hub and Competence Center, Department of Medical Biotechnologies, University of Siena, Italy; Dept of Medical Sciences, Infectious and Tropical Diseases Unit, Azienda Ospedaliera Universitaria Senese, Siena, Italy), Mario Tumbarello (Med Biotech Hub and Competence Center, Department of Medical Biotechnologies, University of Siena, Italy; Dept of Medical Sciences, Infectious and Tropical Diseases Unit, Azienda Ospedaliera Universitaria Senese, Siena, Italy), Ilaria Rancan (Dept of Medical Sciences, Infectious and Tropical Diseases Unit, Azienda Ospedaliera Universitaria Senese, Siena, Italy), Laura Di Sarno (Med Biotech Hub and Competence Center, Department of Medical Biotechnologies, University of Siena, Italy; Medical Genetics, Department of Medical Biotechnologies, University of Siena, Italy), Maria Palmieri (Med Biotech Hub and Competence Center, Department of Medical Biotechnologies, University of Siena, Italy; Medical Genetics, Department of Medical Biotechnologies, University of Siena, Italy), Miriam Lucia Carriero (Med Biotech Hub and Competence Center, Department of Medical Biotechnologies, University of Siena, Italy; Medical Genetics, Department of Medical Biotechnologies, University of Siena, Italy), Massimiliano Fabbiani (Dept of Medical Sciences, Infectious and Tropical Diseases Unit, Azienda Ospedaliera Universitaria Senese, Siena, Italy), Barbara Rossetti (Dept of Medical Sciences, Infectious and Tropical Diseases Unit, Azienda Ospedaliera Universitaria Senese, Siena, Italy), Elena Bargagli (Unit of Respiratory Diseases and Lung Transplantation, Department of Internal and Specialist Medicine, University of Siena), Laura Bergantini (Unit of Respiratory Diseases and Lung Transplantation, Department of Internal and Specialist Medicine, University of Siena), Miriana D’Alessandro (Unit of Respiratory Diseases and Lung Transplantation, Department of Internal and Specialist Medicine, University of Siena), Paolo Cameli (Unit of Respiratory Diseases and Lung Transplantation, Department of Internal and Specialist Medicine, University of Siena), David Bennett (Unit of Respiratory Diseases and Lung Transplantation, Department of Internal and Specialist Medicine, University of Siena), Federico Anedda (Dept of Emergency and Urgency, Medicine, Surgery and Neurosciences, Unit of Intensive Care Medicine, Siena University Hospital, Italy), Simona Marcantonio (Dept of Emergency and Urgency, Medicine, Surgery and Neurosciences, Unit of Intensive Care Medicine, Siena University Hospital, Italy), Sabino Scolletta (Dept of Emergency and Urgency, Medicine, Surgery and Neurosciences, Unit of Intensive Care Medicine, Siena University Hospital, Italy), Federico Franchi (Dept of Emergency and Urgency, Medicine, Surgery and Neurosciences, Unit of Intensive Care Medicine, Siena University Hospital, Italy), Maria Antonietta Mazzei (Department of Medical, Surgical and Neurosciences and Radiological Sciences, Unit of Diagnostic Imaging, University of Siena), Susanna Guerrini (Department of Medical, Surgical and Neurosciences and Radiological Sciences, Unit of Diagnostic Imaging, University of Siena), Edoardo Conticini (Rheumatology Unit, Department of Medicine, Surgery and Neurosciences, University of Siena, Policlinico Le Scotte, Italy), Luca Cantarini (Rheumatology Unit, Department of Medicine, Surgery and Neurosciences, University of Siena, Policlinico Le Scotte, Italy), Bruno Frediani (Rheumatology Unit, Department of Medicine, Surgery and Neurosciences, University of Siena, Policlinico Le Scotte, Italy), Danilo Tacconi (Department of Specialized and Internal Medicine, Infectious Diseases Unit, San Donato Hospital Arezzo, Italy), Chiara Spertilli Raffaelli (Department of Specialized and Internal Medicine, Infectious Diseases Unit, San Donato Hospital Arezzo, Italy), Marco Feri (Dept of Emergency, Anesthesia Unit, San Donato Hospital, Arezzo, Italy), Alice Donati (Dept of Emergency, Anesthesia Unit, San Donato Hospital, Arezzo, Italy), Raffaele Scala (Department of Specialized and Internal Medicine, Pneumology Unit and UTIP, San Donato Hospital, Arezzo, Italy), Luca Guidelli (Department of Specialized and Internal Medicine, Pneumology Unit and UTIP, San Donato Hospital, Arezzo, Italy), Genni Spargi (Department of Emergency, Anesthesia Unit, Misericordia Hospital, Grosseto, Italy), Marta Corridi (Department of Emergency, Anesthesia Unit, Misericordia Hospital, Grosseto, Italy), Cesira Nencioni (Department of Specialized and Internal Medicine, Infectious Diseases Unit, Misericordia Hospital, Grosseto, Italy), Leonardo Croci (Department of Specialized and Internal Medicine, Infectious Diseases Unit, Misericordia Hospital, Grosseto, Italy), Gian Piero Caldarelli (Laboratory Medicine Department, Misericordia Hospital, Grosseto, Italy), Maurizio Spagnesi (Department of Preventive Medicine, Azienda USL Toscana Sud Est, Italy), Paolo Piacentini (Department of Preventive Medicine, Azienda USL Toscana Sud Est, Italy), Maria Bandini (Department of Preventive Medicine, Azienda USL Toscana Sud Est, Italy), Elena Desanctis (Department of Preventive Medicine, Azienda USL Toscana Sud Est, Italy), Silvia Cappelli (Department of Preventive Medicine, Azienda USL Toscana Sud Est, Italy), Anna Canaccini (Territorial Scientific Technician Department, Azienda USL Toscana Sud Est, Italy), Agnese Verzuri (Territorial Scientific Technician Department, Azienda USL Toscana Sud Est, Italy), Valentina Anemoli (Territorial Scientific Technician Department, Azienda USL Toscana Sud Est, Italy), Agostino Ognibene (Laboratory Medicine Department, San Donato Hospital, Arezzo, Italy), Alessandro Pancrazi (Laboratory Medicine Department, San Donato Hospital, Arezzo, Italy), Maria Lorubbio (Laboratory Medicine Department, San Donato Hospital, Arezzo, Italy), Massimo Vaghi (Chirurgia Vascolare, Ospedale Maggiore di Crema, Italy), Antonella D’Arminio Monforte (Department of Health Sciences, Clinic of Infectious Diseases, ASST Santi Paolo e Carlo, University of Milan, Italy), Federica Gaia Miraglia (Department of Health Sciences, Clinic of Infectious Diseases, ASST Santi Paolo e Carlo, University of Milan, Italy), Mario U. Mondelli (Division of Infectious Diseases and Immunology, Fondazione IRCCS Policlinico San Matteo, Pavia, Italy; Department of Internal Medicine and Therapeutics, University of Pavia, Italy), Raffaele Bruno (Division of Infectious Diseases and Immunology, Fondazione IRCCS Policlinico San Matteo, Pavia, Italy; Department of Internal Medicine and Therapeutics, University of Pavia, Italy), Vecchia Marco (Division of Infectious Diseases and Immunology, Fondazione IRCCS Policlinico San Matteo, Pavia, Italy), Stefania Mantovani (Division of Infectious Diseases and Immunology, Fondazione IRCCS Policlinico San Matteo, Pavia, Italy), Serena Ludovisi (Division of Infectious Diseases and Immunology, Fondazione IRCCS Policlinico San Matteo, Pavia, Italy; Department of Internal Medicine and Therapeutics, University of Pavia, Italy), Massimo Girardis (Department of Anesthesia and Intensive Care, University of Modena and Reggio Emilia, Modena, Italy), Sophie Venturelli (Department of Anesthesia and Intensive Care, University of Modena and Reggio Emilia, Modena, Italy), Stefano Busani (Department of Anesthesia and Intensive Care, University of Modena and Reggio Emilia, Modena, Italy), Andrea Cossarizza (Department of Medical and Surgical Sciences for Children and Adults, University of Modena and Reggio Emilia, Modena, Italy), Andrea Antinori (HIV/AIDS Department, National Institute for Infectious Diseases, IRCCS, Lazzaro Spallanzani, Rome, Italy), Alessandra Vergori (HIV/AIDS Department, National Institute for Infectious Diseases, IRCCS, Lazzaro Spallanzani, Rome, Italy), Arianna Emiliozzi (HIV/AIDS Department, National Institute for Infectious Diseases, IRCCS, Lazzaro Spal-lanzani, Rome, Italy), Stefano Rusconi (III Infectious Diseases Unit, ASST-FBF-Sacco, Milan, Italy; Department of Biomedical and Clinical Sciences Luigi Sacco, University of Milan, Milan, Italy), Matteo Siano (Department of Biomedical and Clinical Sciences Luigi Sacco, University of Milan, Milan, Italy), Arianna Gabrieli (Depart-ment of Biomedical and Clinical Sciences Luigi Sacco, University of Milan, Milan, Italy), Agostino Riva (III Infectious Diseases Unit, ASST-FBF Sacco, Milan, Italy; Department of Biomedical and Clinical Sciences Luigi Sacco, University of Milan, Milan, Italy), Daniela Francisci (Infectious Diseases Clinic, Department of Medicine, Azienda Ospedaliera di Perugia and University of Perugia, Santa Maria Hospital, Perugia, Italy; Infectious Diseases Clinic, “Santa Maria” Hospital, University of Perugia, Perugia, Italy), Elisabetta Schiaroli (Infectious Diseases Clinic, Department of Medicine, Azienda Ospedaliera di Perugia and University of Perugia, Santa Maria Hospital, Perugia, Italy), Andrea Tommasi (Infectious Diseases Clinic, Department of Medicine, Azienda Ospedaliera di Perugia and University of Perugia, Santa Maria Hospital, Perugia, Italy), Francesco Paciosi (Infectious Diseases Clinic, Department of Medicine, Azienda Ospedaliera di Perugia and University of Perugia, Santa Maria Hospital, Perugia, Italy), Pier Giorgio Scotton (Department of Infectious Diseases, Treviso Hospital, Local Health Unit 2 Marca Trevigiana, Treviso, Italy), Francesca Andretta (Department of Infectious Diseases, Treviso Hospital, Local Health Unit 2 Marca Trevigiana, Treviso, Italy), Sandro Panese (Clinical Infectious Diseases, Mestre Hospital, Venezia, Italy), Renzo Scaggiante (Infectious Diseases Clinic, ULSS1, Belluno, Italy), Francesca Gatti (Infectious Diseases Clinic, ULSS1, Belluno, Italy), Saverio Giuseppe Parisi (Department of Molecular Medicine, University of Padova, Italy), Melania degli Antoni (Department of Infectious and Tropical Diseases, University of Brescia and ASST Spedali Civili Hospital, Brescia, Italy), Matteo Della Monica (Medical Genetics and Laboratory of Medical Genetics Unit, A.O.R.N. “Antonio Cardarelli”, Naples, Italy), Carmelo Piscopo (Medical Genetics and Laboratory of Medical Genetics Unit, A.O.R.N. “Antonio Cardarelli”, Naples, Italy), Mario Capasso (Department of Molecular Medicine and Medical Biotechnology, University of Naples Federico II, Naples, Italy; CEINGE Biotecnologie Avanzate, Naples, Italy; IRCCS SDN, Naples, Italy), Roberta Russo (Department of Molecular Medicine and Medical Biotechnology, University of Naples Federico II, Naples, Italy; CEINGE Biotecnologie Avanzate, Naples, Italy), Immacolata Andolfo (Department of Molecular Medicine and Medical Biotechnology, University of Naples Federico II, Naples, Italy; CEINGE Biotecnologie Avanzate, Naples, Italy), Achille Iolascon (Department of Molecular Medicine and Medical Biotechnology, University of Naples Federico II, Naples, Italy; CEINGE Biotecnologie Avanzate, Naples, Italy), Giuseppe Fiorentino (Unit of Respiratory Physiopathology, AORN dei Colli, Monaldi Hospital, Naples, Italy), Massimo Carella (Division of Medical Genetics, Fondazione IRCCS Casa Sollievo della Sofferenza Hospital, San Giovanni Rotondo, Italy), Marco Castori (Division of Medical Genetics, Fondazione IRCCS Casa Sollievo della Sofferenza Hospital, San Giovanni Rotondo, Italy), Giuseppe Merla (Division of Medical Genetics, Fondazione IRCCS Casa Sollievo della Sofferenza Hospital, San Giovanni Rotondo, Italy), Gabriella Maria Squeo (Division of Medical Genetics, Fondazione IRCCS Casa Sollievo della Sofferenza Hospital, San Giovanni Rotondo, Italy), Filippo Aucella (Department of Medical Sciences, Fondazione IRCCS Casa Sollievo della Sofferenza Hospital, San Giovanni Rotondo, Italy), Pamela Raggi (Clinical Trial Office, Fondazione IRCCS Casa Sollievo della Sofferenza Hospital, San Giovanni Rotondo, Italy), Carmen Marciano (Clinical Trial Office, Fondazione IRCCS Casa Sollievo della Sofferenza Hospital, San Giovanni Rotondo, Italy), Rita Perna (Clinical Trial Office, Fondazione IRCCS Casa Sollievo della Sofferenza Hospital, San Giovanni Rotondo, Italy), Matteo Bassetti (Department of Health Sciences, University of Genova, Genova, Italy; Infectious Diseases Clinic, Policlinico San Martino Hospital, IRCCS for Cancer Research Genova, Italy), Antonio Di Biagio (Department of Health Sciences, University of Genova, Genova, Italy; Infectious Diseases Clinic, Policlinico San Martino Hospital, IRCCS for Cancer Research Genova, Italy), Maurizio Sanguinetti (Microbiology, Fondazione Policlinico Universitario Agostino Gemelli IRCCS, Catholic University of Medicine, Rome, Italy; Department of Laboratory Sciences and Infectious Diseases, Fondazione Policlinico Universitario A. Gemelli IRCCS, Rome, Italy), Luca Masucci (Microbiology, Fondazione Policlinico Universitario Agostino Gemelli IRCCS, Catholic University of Medicine, Rome, Italy; Department of Laboratory Sciences and Infectious Diseases, Fondazione Policlinico Universitario A. Gemelli IRCCS, Rome, Italy), Serafina Valente (Department of Cardiovascular Diseases, University of Siena, Siena, Italy), Marco Mandalà (Otolaryngology Unit, University of Siena, Italy), Alessia Giorli (Otolaryngology Unit, University of Siena, Italy), Lorenzo Salerni (Otolaryngology Unit, University of Siena, Italy), Patrizia Zucchi (Department of Internal Medicine, ASST Valtellina e Alto Lario, Sondrio, Italy), Pierpaolo Parravicini (Department of Internal Medicine, ASST Valtellina e Alto Lario, Sondrio, Italy), Elisabetta Menatti (Study Coordinator Oncologia Medica e Ufficio Flussi, Sondrio, Italy), Stefano Baratti (Clinical Infectious Diseases, Mestre Hospital, Venezia, Italy), Tullio Trotta (First Aid Department, Luigi Curto Hospital, Polla, Salerno, Italy), Ferdinando Giannattasio (First Aid Department, Luigi Curto Hospital, Polla, Salerno, Italy), Gabriella Coiro (First Aid Department, Luigi Curto Hospital, Polla, Salerno, Italy), Fabio Lena (Local Health Unit-Pharmaceutical Department of Grosseto, Toscana Sud Est Local Health Unit, Grosseto, Italy), Domenico A. Coviello (U.O.C. Laboratorio di Genetica Umana, IRCCS Istituto G. Gaslini, Genova, Italy), Cristina Mussini (Infectious Diseases Clinics, University of Modena and Reggio Emilia, Modena, Italy), Giancarlo Bosio (Department of Respiratory Diseases, Azienda Ospedaliera di Cremona, Cremona, Italy), Enrico Martinelli (Department of Respiratory Diseases, Azienda Ospedaliera di Cremona, Cremona, Italy), Sandro Mancarella (U.O.C. Medicina, ASST Nord Milano, Ospedale Bassini, Cinisello Balsamo (MI), Italy), Luisa Tavecchia (U.O.C. Medicina, ASST Nord Milano, Ospedale Bassini, Cinisello Balsamo (MI), Italy), Mary Ann Belli (U.O.C. Medicina, ASST Nord Milano, Ospedale Bassini, Cinisello Balsamo (MI), Italy), Lia Crotti (Istituto Auxologico Italiano, IRCCS, Department of Cardiovascular, Neural and Metabolic Sciences, San Luca Hospital, Milan, Italy; Department of Medicine and Surgery, University of Milano-Bicocca, Milan, Italy; Istituto Auxologico Italiano, IRCCS, Center for Cardiac Arrhythmias of Genetic Origin, Milan, Italy; Istituto Auxologico Italiano, IRCCS, Laboratory of Cardiovascular Genetics, Milan, Italy; Member of the European Reference Network for Rare, Low Prevalence and Complex Diseases of the Heart-ERN GUARD-Heart), Gianfranco Parati (Istituto Auxologico Italiano, IRCCS, Department of Cardiovascular, Neural and Metabolic Sciences, San Luca Hospital, Milan, Italy; Department of Medicine and Surgery, University of Milano-Bicocca, Milan, Italy), Nicola Picchiotti (University of Siena, DIISM-SAILAB, Siena, Italy; Department of Mathematics, University of Pavia, Pavia, Italy), Marco Gori (University of Siena, DIISM-SAILAB, Siena, Italy; University Cote d’Azur, Inria, CNRS, I3S, Maasai), Chiara Gabbi (Independent Medical Scientist, Milan, Italy), Maurizio Sanarico (Independent Data Scientist, Milan, Italy), Stefano Ceri (Department of Electronics, Information and Bioengineering (DEIB), Politecnico di Milano, Milano, Italy), Pietro Pinoli (Department of Electronics, Information and Bioengineering (DEIB), Politecnico di Milano, Milano, Italy), Francesco Raimondi (Scuola Normale Superiore, Pisa, Italy), Filippo Biscarini (CNR-Consiglio Nazionale delle Ricerche, Istituto di Biologia e Biotecnologia Agraria (IBBA), Milano, Italy), Alessandra Stella (CNR-Consiglio Nazionale delle Ricerche, Istituto di Biologia e Biotecnologia Agraria (IBBA), Milano, Italy), Marco Rizzi (Unit of Infectious Diseases, ASST Papa Giovanni XXIII Hospital, Bergamo, Italy), Franco Maggiolo (Unit of Infectious Diseases, ASST Papa Giovanni XXIII Hospital, Bergamo, Italy), Diego Ripamonti (Unit of Infectious Diseases, ASST Papa Giovanni XXIII Hospital, Bergamo, Italy), Claudia Suardi (Fondazione per la ricerca Ospedale di Bergamo, Bergamo, Italy), Tiziana Bachetti (Direzione Scientifica, Istituti Clinici Scientifici Maugeri IRCCS, Pavia, Italy), Maria Teresa La Rovere (Istituti Clinici Scientifici Maugeri IRCCS, Department of Cardiology, Institute of Montescano, Pavia, Italy), Simona Sarzi-Braga (Istituti Clinici Scientifici Maugeri, IRCCS, Department of Cardiac Rehabilitation, Institute of Tradate (VA), Italy), Maurizio Bussotti (Istituti Clinici Scientifici Maugeri IRCCS, Department of Cardiology, Institute of Milan, Milan, Italy), Katia Capitani (Med Biotech Hub and Competence Center, Department of Medical Biotechnologies, University of Siena, Siena, Italy; Core Research Laboratory, ISPRO, Florence, Italy), Kristina Zguro (Med Biotech Hub and Competence Center, Department of Medical Biotechnologies, University of Siena, Siena, Italy), Simona Dei (Health Management, Azienda USL Toscana Sudest, Tuscany, Italy), Sabrina Ravaglia (IRCCS C. Mondino Foundation, Pavia, Italy), Rosangela Artuso (Medical Genetics Unit, Meyer Children’s University Hospital, Florence, Italy), Antonio Perrella (Department of Medicine, Pneumology Unit, Misericordia Hospital, Grosseto, Italy), Francesco Bianchi (Med Biotech Hub and Competence Center, Department of Medical Biotechnologies, University of Siena, Siena, Italy; Department of Medicine, Pneumology Unit, Misericordia Hospital, Grosseto, Italy), Paola Bergomi (Department of Anesthesia and Intensive Care Unit, ASST Fatebenefratelli Sacco, Luigi Sacco Hospital, Polo Universitario, University of Milan, Milan), Emanuele Catena (Department of Anesthesia and Intensive Care Unit, ASST Fatebenefratelli Sacco, Luigi Sacco Hospital, Polo Universitario, University of Milan, Milan), Riccardo Colombo (Department of Anesthesia and Intensive Care Unit, ASST Fatebenefratelli Sacco, Luigi Sacco Hospital, Polo Universitario, University of Milan, Milan), Valentina Perticaroli (Med Biotech Hub and Competence Center, Department of Medical Biotechnologies, University of Siena, Siena, Italy; Medical Genetics, Department of Medical Biotechnologies, University of Siena, Italy; Genetica Medica, Azienda Ospedaliero-Universitaria Senese, Italy).
**Collaborators of GEVACOBA Study Group**
  Massimo Gennarelli (Department of Molecular and Translational Medicine, University of Brescia, Brescia, Italy; Genetics Unit, IRCCS Istituto Centro San Giovanni di Dio Fatebenefratelli, Brescia, Italy), Chiara Magri (Department of Molecular and Translational Medicine, University of Brescia, Brescia, Italy), Giorgio Basiotto (Department of Molecular and Translational Medicine, University of Brescia, Brescia, Italy; Clinical Chemistry Laboratory, Cytogenetics and Molecular Genetics Section, Diagnostic Department, ASST Spedali Civili di Brescia, Brescia, Italy), Daniela Zizioli (Department of Molecular and Translational Medicine, University of Brescia, Brescia, Italy), Silvia Giliani (Department of Molecular and Translational Medicine, University of Brescia, Brescia, Italy; Clinical Chemistry Laboratory, Cytogenetics and Molecular Genetics Section, Diagnostic Department, ASST Spedali Civili di Brescia, Brescia, Italy; Angelo Nocivelli Institute for Molecular Medicine, Brescia, Italy), Eugenio Monti (Department of Molecular and Translational Medicine, University of Brescia, Brescia, Italy), Emanuele Focà (Department of Clinical and Experimental Sciences, University of Brescia, Brescia, Italy; Division of Infectious and Tropical Diseases, ASST Spedali Civili di Brescia, Brescia, Italy), Cannio Carriero (Department of Clinical and Experimental Sciences, University of Brescia, Brescia, Italy; Division of Infectious and Tropical Diseases, ASST Spedali Civili di Brescia, Brescia, Italy), Nicola Latronico (University Division of Anesthesiology and Critical Care Medicine, ASST Spedali Civili, 25123 Brescia, Italy; Department of Medical and Surgical Specialties, Radiological Sciences and Public Health, University of Brescia, Brescia, Italy), Alessandro Padovani (Department of Clinical and Experimental Sciences, University of Brescia, Brescia, Italy; Neurology Unit, ASST Spedali Civili di Brescia), Duilio Brugnoni (Clinical Chemistry Laboratory, Diagnostic Department, ASST Spedali Civili di Brescia).
